# Supplementary material for: HIV-1 DNA predicts disease progression and post-treatment virological control
Source: eLife. 2014 Sep 12;3:e03821. doi: 10.7554/eLife.03821 (PMC4199415; doi:10.7554/eLife.03821)
Supplement: Supplementary file 1. — Additional demographics of randomized participants included in untreated and 48 week short-course ART analyses Demographics of participants available for analyses of those randomised to receive either no therapy from PHI (first column) and those randomised to receive 48 of weeks of ART from PHI (second column). Data as indicated were: † determined at pre-therapy baseline (trial week 0), * determined at week 48, prior to TI or + median (interquartile range). SOC: Standard of Care trial arm. DOI: http://dx.doi.org/10.7554/eLife.03821.014 [file elife03821s001.docx]

**Supplementary file 1**

**Additional demographics of randomized participants included in untreated and 48 week short-course ART analyses**

|  | **Randomised to no therapy (SOC) at PHI, and sampled at week 0**  **N = 51** | **Randomised to 48 weeks of ART (ART-48) from PHI and sampled at week 48**  **N=47** |
| --- | --- | --- |
| **Patients with a Total HIV-1 DNA measurement** | 51 (100%)^†^ | 47 (100%)* |
| **Patients with an Integrated HIV-1 DNA measurement** | 38 (74%)^†^ | 47 (100%)* |
| **Total HIV-1 DNA**  **(log_10_ copies/million CD4 cells)^+^** | 4.01 (3.49-4.29) ^†^ | 3.25 (2.90-3.44)* |
| **Integrated HIV-1 DNA**  **(log_10_ copies/million CD4 cells) ^+^** | 3.6 (3.3-3.8) ^†^ | 3.0 (2.7-3.2)* |
| **Time since seroconversion at enrolment (days) ^+^** | 73 (49-93) | 76.5 (52.0-96.9) |
| **ART Duration (weeks)^+^** | 0 | 48 (47.7 – 48.7) |
| **Baseline CD4 cell count**  **(cells/µl) ^+^** | 550 (400 – 691) | 565 (439-732) |
| **Log_10_ RNA copies/ml^+^** | 4.73 (4.14 – 5.26) | All <50 |
| **Country of recruitment** | Australia 8 (15.6%)  Italy 7 (13.7%)  Brazil 5 (9.8%)  UK 31 (60.8%) | Australia 5 (10.6%)  Italy 5 (10.6%)  Brazil 3 (6.4%)  UK 34 (72.3%) |
| **Sex** | Female 2 (4.0%)  Male 49 (96%) | Female 1 (1.9%)  Male 51 (98%) |

^†^ determined at pre-therapy baseline (trial week 0). * determined at week 48, prior to TI. **^+^**median (interquartile range). SOC: Standard of Care trial arm.
