# Supplementary material for: HIV-1 DNA predicts disease progression and post-treatment virological control
Source: eLife. 2014 Sep 12;3:e03821. doi: 10.7554/eLife.03821 (PMC4199415; doi:10.7554/eLife.03821)
Supplement: Supplementary file 2. — Sample numbers available at each time-point by trial randomization. Numbers of samples available at each time-point are presented. Participants from all three trial arms were included at week 0 as they were all treatment naïve at this point. Not all patients at any one time-point are always represented at other time-points due to variation in sample availability. Trial arms: SOC: Standard of Care (untreated); ART-48: 48 weeks of ART after randomization; ART-12: 12 weeks of ART after randomization. DOI: http://dx.doi.org/10.7554/eLife.03821.015 [file elife03821s002.docx]

**Supplementary file 2**

**Sample numbers available at each time-point by trial randomization.**

| Time post randomization (weeks) | **SOC** | | **ART-48** | | **ART-12** | |
| --- | --- | --- | --- | --- | --- | --- |
|  | Total (n) | Integrated (n) | Total (n) | Integrated (n) | Total (n) | Integrated (n) |
| 0 | 51 | 38 | 52 | 35 | 51 | 38 |
| 48 | - | - | 47 | 47 | - | - |
| 52 | - | - | 46 | 39 | - | - |
| 60 | - | - | 32 | 27 | - | - |
| 108 | - | - | 48 | 31 | - | - |

Numbers of samples available at each time-point are presented. Participants from all three trial arms were included at week as they were all treatment naïve at this point. Not all patients at any one time-point are always represented at other time-points due to variation in sample availability. Trial arms: SOC: Standard of Care (untreated); ART-48: 48 weeks of ART after randomization; ART-12: 12 weeks of ART after randomization.
