# Supplementary material for: HIV-1 DNA predicts disease progression and post-treatment virological control
Source: eLife. 2014 Sep 12;3:e03821. doi: 10.7554/eLife.03821 (PMC4199415; doi:10.7554/eLife.03821)
Supplement: Supplementary file 3. — Cox regression models for variables associated with time to rebound of 400 copies/ml and sampled at wk48. Table to show results of Cox regression analysis for time to virological rebound of 400 copies/ml of plasma with Total DNA and CD4 T cell count as covariables. Univariable and multivariable data are presented with Hazard Ratios (HR) with 95% Confidence Intervals (CI) and associated P values. DOI: http://dx.doi.org/10.7554/eLife.03821.016 [file elife03821s003.docx]

**Supplementary file 3**

**Cox regression models for variables associated with time to rebound of 400 copies/ml and sampled at wk48**

| **Univariable unadjusted** | | **Multivariable adjusted** | |  |
| --- | --- | --- | --- | --- |
| **Covariate** | **HR (95% CI)** | **P value** | **HR (95% CI)** | **P value** |
| Total DNA (log_10_ DNA copies) | 2.43 (1.23-4.79) | 0.010 | 2.68 (1.31-5.48) | 0.0069 |
| CD4+ T cell count / 100 cells | 0.92 (0.78-1.08) | 0.32 | 1.06 (0.95-1.20) | 0.26 |
