# Supplementary material for: HIV-1 DNA predicts disease progression and post-treatment virological control
Source: eLife. 2014 Sep 12;3:e03821. doi: 10.7554/eLife.03821 (PMC4199415; doi:10.7554/eLife.03821)
Supplement: Supplementary file 4. — 2 × 2 table comparing the number of patients with different times to 50 and 400 copy/ml rebound and their total and integrated pre-TI HIV-1 DNA levels. Table to compare the association between HIV-1 DNA levels at TI (both Total and Integrated) and time to a plasma viral load of either between 50–400 copies/ml or greater than 400 copies/ml. HIV-1 DNA levels were split into ‘high’ and ‘low’ by the median value. The proportions are significantly different by Fisher's exact test for Total (p = 0.0074) but not Integrated HIV-1-DNA levels (p = 0.091). DOI: http://dx.doi.org/10.7554/eLife.03821.017 [file elife03821s004.docx]

**Supplementary file 4**

**2x2 table comparing the number of patients with different times to 50 and 400 copy/ml rebound and their total and integrated pre-TI HIV-1 DNA levels**

|  | **High Integrated HIV-1-DNA** | **Low Integrated HIV-1-DNA** |
| --- | --- | --- |
| First detectable VL rebound >50 and <400 copies/ml | N=3 | N=9 |
| First detectable VL rebound >400 copies/ml | N=19 | N=14 |
|  |  |  |
|  | **High Total HIV-1-DNA** | **Low Total HIV-1-DNA** |
| First detectable VL rebound >50 and <400 copies/ml | N=2 | N=10 |
| First detectable VL rebound >400 copies/ml | N=21 | N=12 |
